# Understanding the Complexities of Community-Dwelling Older Adults’ Lived Experiences During COVID-19

**DOI:** 10.1093/geroni/igab046.052

**Published:** 2021-12-17

**Authors:** Bo Xie, Kristina Shiroma, Atami Sagna de Main, Nathan Davis, Karen Fingerman, Valerie Danesh

**Affiliations:** 1 The University of Texas at Austin, Austin, Texas, United States; 2 The University of Texas at Austin, The University of Texas at Austin, Texas, United States

## Abstract

Since December 2019, COVID-19 has spurred rapid and extensive research, but this research has focused on some perspectives with others understudied. In particular, studies have not yet explored the complexities of community-dwelling older adults’ lived experiences during the pandemic. This study aimed to address this gap. Community-dwelling older adults living in Central Texas (N = 200; age, 65–92 years, M = 73.6± 6.33) responded to open- and closed-ended questions over the telephone during June–August 2020. Data were analyzed using inductive thematic analysis. We identified three key themes. (1) Positive experiences, with 4 subthemes: perception that the pandemic has not changed one’s lifestyle; adjusting well—particularly with the aid of technology; being positive in perspective; and a “loner advantage” (being a “loner” pre-pandemic was advantageous during the pandemic). (2) Mixed experiences, with 4 subthemes: doing okay but unhappy about changing lifestyle routines; doing okay but unhappy about loss of in-person interactions with family and friends; doing okay but frustrated by witnessing absence of social distancing or facemask use by others; and maintaining physical health with fluctuating symptoms of depression or anxiety. (3) Negative experiences, with 3 subthemes: bitter about others/society/government not caring for older adults; feeling isolated, bored, and powerless; and worsening as time goes by. A thematic map was subsequently developed. These findings reveal the complexities of community-dwelling older adults’ lived experiences, illustrating effective coping and resilience during the pandemic and dissatisfaction owing to the pandemic’s effects on their lives and to their observations of others’ behaviors.

